# COVID-19-Forschungsdaten leichter zugänglich machen – Aufbau einer bundesweiten Informationsinfrastruktur

**DOI:** 10.1007/s00103-021-03386-x

**Published:** 2021-07-23

**Authors:** Carsten Oliver Schmidt, Juliane Fluck, Martin Golebiewski, Linus Grabenhenrich, Horst Hahn, Toralf Kirsten, Sebastian Klammt, Matthias Löbe, Ulrich Sax, Sylvia Thun, Iris Pigeot, NFDI4Health Task Force Covid-19, Wolfgang Ahrens, Johannes Darms, Jörg Henke, Xiaoming Hu, Sophie Klopfenstein, Lisa Langnickel, Bianca Lassen-Schmidt, Hermann Pohlabeln, Michael Lieser, Anatol-Fiete Näher, Markus Scholz, Carina Vorisek, Dagmar Waltemath, Hannes Wünsche

**Affiliations:** 1 Institut für Community Medicine, Universitätsmedizin Greifswald, Walther-Rathenau-Str. 48, 17475 Greifswald, Deutschland; 2 ZB MED – Informationszentrum Lebenswissenschaften, Bonn, Deutschland; 3 Institut für Geodäsie und Geoinformation, Rheinische Friedrich-Wilhelms-Universität Bonn, Bonn, Deutschland; 4 Abteilung Bioinformatik, Fraunhofer Institut SCAI, Sankt Augustin, Deutschland; 5 Heidelberger Institut für Theoretische Studien (HITS), Heidelberg, Deutschland; 6 Robert Koch-Institut, Berlin, Deutschland; 7 Institut für Digitale Medizin, Fraunhofer MEVIS, Bremen, Deutschland; 8 Jacobs University, Bremen, Deutschland; 9 Fakultät Angewandte Computer- und Biowissenschaften, Hochschule Mittweida, Mittweida, Deutschland; 10 Institut für Medical Data Science, Universitätsmedizin Leipzig, Leipzig, Deutschland; 11 Netzwerk der Koordinierungszentren für Klinische Studien – KKS-Netzwerk e. V., Berlin, Deutschland; 12 Institut für Medizinische Informatik, Statistik und Epidemiologie (IMISE), Universität Leipzig, Leipzig, Deutschland; 13 Institut für Medizinische Informatik, Universitätsmedizin Göttingen, Göttingen, Deutschland; 14 Berlin Institute of Health at Charité, Universitätsmedizin Berlin, Berlin, Deutschland; 15 Leibniz-Institut für Präventionsforschung und Epidemiologie – BIPS, Bremen, Deutschland; 16 Fachbereich Mathematik und Informatik, Universität Bremen, Bremen, Deutschland

**Keywords:** COVID-19-Studienportal, FAIR-Prinzipien, Epidemiologie, Public Health, Klinische Studien, COVID-19 study portal, FAIR principles, Epidemiology, Public health, Clinical trials

## Abstract

Public-Health-Forschung, epidemiologische und klinische Studien sind erforderlich, um die COVID-19-Pandemie besser zu verstehen und geeignete Maßnahmen zu ergreifen. Daher wurden auch in Deutschland zahlreiche Forschungsprojekte initiiert. Zum heutigen Zeitpunkt ist es ob der Fülle an Informationen jedoch kaum noch möglich, einen Überblick über die vielfältigen Forschungsaktivitäten und deren Ergebnisse zu erhalten. Im Rahmen der Initiative „Nationale Forschungsdateninfrastruktur für personenbezogene Gesundheitsdaten“ (NFDI4Health) schafft die „Task Force COVID-19“ einen leichteren Zugang zu SARS-CoV-2- und COVID-19-bezogenen klinischen, epidemiologischen und Public-Health-Forschungsdaten. Dabei werden die sogenannten FAIR-Prinzipien (Findable, Accessible, Interoperable, Reusable) berücksichtigt, die eine schnellere Kommunikation von Ergebnissen befördern sollen. Zu den wesentlichen Arbeitsinhalten der Taskforce gehören die Erstellung eines Studienportals mit Metadaten, Erhebungsinstrumenten, Studiendokumenten, Studienergebnissen und Veröffentlichungen sowie einer Suchmaschine für Preprint-Publikationen. Weitere Inhalte sind ein Konzept zur Verknüpfung von Forschungs- und Routinedaten, Services zum verbesserten Umgang mit Bilddaten und die Anwendung standardisierter Analyseroutinen für harmonisierte Qualitätsbewertungen. Die im Aufbau befindliche Infrastruktur erleichtert die Auffindbarkeit von und den Umgang mit deutscher COVID-19-Forschung. Die im Rahmen der NFDI4Health Task Force COVID-19 begonnenen Entwicklungen sind für weitere Forschungsthemen nachnutzbar, da die adressierten Herausforderungen generisch für die Auffindbarkeit von und den Umgang mit Forschungsdaten sind.

## Abstract (en)

Public health research and epidemiological and clinical studies are necessary to understand the COVID-19 pandemic and to take appropriate action. Therefore, since early 2020, numerous research projects have also been initiated in Germany. However, due to the large amount of information, it is currently difficult to get an overview of the diverse research activities and their results. Based on the “Federated research data infrastructure for personal health data” (NFDI4Health) initiative, the “COVID-19 task force” is able to create easier access to SARS-CoV-2- and COVID-19-related clinical, epidemiological, and public health research data. Therefore, the so-called FAIR data principles (findable, accessible, interoperable, reusable) are taken into account and should allow an expedited communication of results. The most essential work of the task force includes the generation of a study portal with metadata, selected instruments, other study documents, and study results as well as a search engine for preprint publications. Additional contents include a concept for the linkage between research and routine data, a service for an enhanced practice of image data, and the application of a standardized analysis routine for harmonized quality assessment. This infrastructure, currently being established, will facilitate the findability and handling of German COVID-19 research. The developments initiated in the context of the NFDI4Health COVID-19 task force are reusable for further research topics, as the challenges addressed are generic for the findability of and the handling with research data.

## Einleitung

COVID-19 stellt Individuen und Gesellschaften weltweit vor eine der größten Herausforderungen der letzten Jahrzehnte. Public-Health-Forschung, epidemiologische und klinische Studien sind daher unabdingbar, um die Ausbreitung des für die Pandemie verantwortlichen SARS-CoV‑2 und dessen Varianten nachzuverfolgen, die Folgen für die Gesundheit und das soziale Leben besser zu verstehen sowie wirksame Therapie- und Impfmethoden zu identifizieren. Dadurch entsteht für Politik, Wirtschaft, Gesundheitsversorgung und Gesellschaft eine empirische Grundlage zur Eindämmung und zum Umgang mit der Pandemie. Diese bedarf jedoch einer fortlaufenden Aktualisierung.

In sehr kurzer Zeit entstanden zahlreiche Projekte, Studien und Netzwerke zur Erforschung von SARS-CoV‑2 und COVID-19. Aus der Perspektive von Forschenden sind hiermit erhebliche Herausforderungen verbunden. Es fällt zunehmend schwer, einen Überblick zu behalten. Dieser Überblick ist jedoch unabdingbar, um Forschungsaktivitäten besser zu koordinieren, ungeplante Doppelforschung zu vermeiden und Studien harmonisiert zu implementieren. Derzeit wird die Zusammenführung von Wissensquellen durch die unzureichende Berücksichtigung von Standards und einen Mangel an harmonisierten Methoden auf allen Ebenen des Forschungsprozesses erschwert.

Aufgrund der bestehenden Pflicht zur Registrierung klinischer Studien in Registern sind deren Metadaten gut strukturiert verfügbar, z. B. in der *International Clinical Trials Registry Platform* (ICTRP) der Weltgesundheitsorganisation (WHO; [1]) oder dem Deutschen Register Klinischer Studien (DRKS; [2]). Dagegen ist die Situation für epidemiologische und Public-Health-Studien wesentlich unübersichtlicher. Zwar gibt es national und international mehrere Übersichten im Internet, z. B. zu seroepidemiologischen Studien am Robert Koch-Institut (RKI; [3]), ein COVID-19-Forschungsregister der *American Society for Microbiology* [4], die COVID-19-Forschungsübersicht der Medizininformatik-Initiative [5] oder des Rats für Sozial- und Wirtschaftsdaten [6], aber diese Übersichten sind in Umfang, Aktualität und Informationstiefe uneinheitlich.

Noch schwieriger wird es, wenn studienübergreifend ein detaillierter Einblick in die Protokolle, Erhebungsinstrumente, Itembanken und weitere Studiendokumente gewonnen werden soll, obwohl Technologien zur übersichtlichen Aufbereitung und Darstellung solcher Informationen grundsätzlich verfügbar sind [7–9]. Dies wäre zum Beispiel wichtig, um eigene Erhebungen mit bestehenden vergleichbar zu planen. Nur vereinzelt bieten Projekte Zugang zu relevanten Informationen. So wurde im Netzwerk Universitätsmedizin (NUM; [10]), das vor allem krankenhausbezogene Forschung koordiniert, mit dem German Corona Consensus Dataset (GECCO) ein positives Beispiel für harmonisierte Datenerhebungen auf Basis von internationalen medizinischen IT-Standards anhand eines abgestimmten Kerndatensatzes geschaffen [11]. Auch das GESIS – Leibniz-Institut für Sozialwissenschaften stellt eigene Erhebungsinstrumente und weitere Studiendokumente auf ihren Webseiten bereit [12].

Ein weiteres Problem betrifft den Zugriff auf neueste Forschungsergebnisse und Daten, die inzwischen häufig in Preprints publiziert werden, bevor sie mit oft mehrmonatigen Verzögerungen in etablierten Literaturdatenbanken wie PubMed oder Web of Science auffindbar sind. Ein zentraler Zugriff auf diese verteilt vorliegenden Preprint-Archive ist daher wichtig, um aktuelle Ergebnisse besser zu finden und die Idee des breiten Communityreviews von Preprints im Sinne einer Qualitätssicherung praktisch zu unterstützen.

Darüber hinaus wurden im Laufe der Pandemie weitere Hürden offensichtlich, die eine effiziente Forschung erschweren: Obwohl teilweise dieselben Personen in verschiedene Studien eingeschlossen wurden und weitere Gesundheitsdaten dieser Personen z. B. bei den Krankenkassen gespeichert sind, fehlen ausreichende Optionen, diese Daten auf individueller Ebene zu verknüpfen. Dies beschränkt die Möglichkeiten, ein ausreichend umfassendes Bild des Krankheitsgeschehens zu erhalten, um verlässlichere Aussagen zur Verlaufsprognose oder zu Impffolgen schnell zu erhalten. Dies ist der Fall, obwohl es außerhalb von reinen Forschungsprojekten positive Beispiele gibt, die durch eine entsprechende Gesetzgebung abgedeckt sind, wie etwa die Zusammenführung von Melde- und Sequenzierungsdaten am RKI, um verbesserte Aussagen zu Virusvarianten zu erhalten.

Zusammenfassend erfüllen die deutschen klinischen Studien zu COVID-19 sowie entsprechende Datenbestände in Epidemiologie und Public Health trotz positiver Beispiele die Ansprüche der sogenannten FAIR-Prinzipien [13] noch nicht im vollen Umfang. Dabei steht FAIR für die Auffindbarkeit (Findable), Zugänglichkeit (Accessible), Interoperabilität (Interoperable) und Wiederverwendbarkeit (Reusable) von Forschungsdaten LINK zu [14]. Um diesem Defizit zu begegnen, wurde als Teil des deutschen interdisziplinären Netzwerkprojekts „Nationale Forschungsdateninfrastruktur für personenbezogene Gesundheitsdaten“ (NFDI4Health; [15]) die Task Force COVID-19 etabliert [16]. Ihr Ziel ist es, eine bundesweite Informationsinfrastruktur zu entwickeln, um medizinische, epidemiologische und Public-Health-Forschung nach FAIR-Kriterien leichter zugänglich zu machen und Forschungsergebnisse besser zu kommunizieren. Dabei betrachtet die NFDI4Health Task Force COVID-19 neben Forschung in Bezug auf PatientInnen mit COVID-19 auch die Public-Health-Folgen des Pandemieausbruchs auf die Allgemeinbevölkerung.

Wesentliche Arbeitsinhalte der NFDI4Health Task Force COVID-19 umfassen:

1. die Erstellung eines Studienportals zum Auffinden deutscher COVID-19-Forschungsinitiativen mit strukturierten Gesundheitsdaten aus epidemiologischen und klinischen Studien inkl. Impfstudien, administrativen Datenbanken, der Primärversorgung und der Gesundheitsberichterstattung, das auch die semantisch aufbereitete vergleichende Darstellung von Items aus Erhebungsinstrumenten umfasst;
2. die auf Text-Mining (Extraktion aus großen Textmengen) basierende Aufbereitung und Darstellung von SARS-CoV-2- und COVID-19-bezogener Forschung unter Nutzung der Inhalte verschiedener Preprint-Server in einer semantischen Suchmaschine;
3. ein Konzept zur Verknüpfung von Forschungs- und Routinedaten;
4. Services zum verbesserten Umgang mit Bilddaten;
5. die Anwendung standardisierter Analyseroutinen für harmonisierte Qualitätsbewertungen.

**Figure 1:**
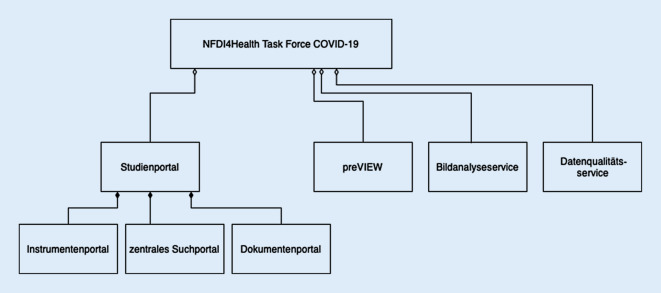

Die primäre Zielgruppe der Infrastruktur sind Forschende, die Studien zu SARS-CoV‑2 oder COVID-19 durchführen oder planen. Die intendierte Anwendung der ersten beiden Arbeitsinhalte betrifft insbesondere das leichtere Auffinden deutscher COVID-19-Ressourcen zur besseren Planung eigener Studien sowie zur Bewertung von Studienergebnissen. Mit den Arbeitsinhalten 3–5 werden Hilfsmittel an die Hand gegeben, um die Qualität spezifischer Aspekte von COVID-19-bezogener Forschung wie das Verknüpfen von Daten aus verschiedenen Quellen (Record-Linkage) oder Bildanalyse zu unterstützen. Eine Übersicht zu den verschiedenen Arbeitsinhalten findet sich in Abb. 1. Der Schwerpunkt dieser Publikation liegt auf der Darstellung des Studienportals und der Suchmaschine für Preprint-Publikationen (Arbeitsinhalte 1–2).

## COVID-19-Studienportal

Um ein Studienportal entwickeln zu können (s. Arbeitsinhalt 1), das einerseits mit Registern für klinische Studien kompatibel ist, insbesondere dem ICTRP [1] und dem DRKS [2], und andererseits epidemiologische und Public-Health-Studien angemessen abbildet, musste ein geeignetes Datenmodell formuliert werden. Mit diesem wird eine strukturierte Erfassung von studienbezogenen Metadaten in einer Datenbank ermöglicht. Zu diesem Zweck greift es auf Attribute und Wertelisten aus einer Reihe von Vorarbeiten zurück, z. B. den *Minimum Information About Biobank Data Sharing* (MIABIS; [17, 18]), das Maelstrom-Datenmodel [19] sowie dem DataCite-Metadatenschema [20]. Ein Mapping gegen die Standards HL7 FHIR [21] und das Clinical Data Interchange Standards Consortium – Operational Data Model (CDISC ODM) wurden umgesetzt, um eine angemessene Interoperabilität zu gewährleisten. HL7 FHIR sowie CDISC ODM sind Standards zum Austausch strukturierter klinischer Daten zwischen Geräten und Organisationen. Da keiner der Standards eine vollständige Abdeckung der Problemdomäne bot, wurde das Datenmodell zunächst nicht konsistent innerhalb eines dieser Standards formuliert. Details des Datenmodells sind in einer eigenen Publikation verfügbar [22]. Durch die Kompatibilität mit dem Datenformat von DataCite können Studiendokumente und Instrumente einzeln publiziert und ein DOI (Document Object Identifier) vergeben werden [23]. So wird auch „graue Literatur“ referenzier- und zitierbar. Darüber hinaus kann eine forschungsfreundliche Lizenz für deren Nachnutzung im Creative Commons Framework [24] vergeben werden.

Um die relationale Datenbank zu befüllen, die das Datenmodell implementiert, wurde im ersten Schritt ein automatisches Verfahren entwickelt, um Einträge zu klinischen Studien aus den Registern DRKS und WHO ICTRP regelmäßig auszulesen. Im zweiten Schritt wurden epidemiologische und Public-Health-Studien anhand geeigneter Suchmaschinen wie Google und PubMed identifiziert, die jeweiligen Studienleitungen individuell kontaktiert und um ihre Teilnahme an dem Studienportal gebeten. Studienmerkmale wurden in diesem Prozess manuell erfasst.

Das COVID-19-Studienportal [25] mit seinen 3 Teilkomponenten zentrales Suchportal, Dokumentenportal und Instrumentenportal bedient verschiedene Anwendungsfälle, die nachfolgend beschrieben sind.

### Zentrales Suchportal

**Figure 2:**
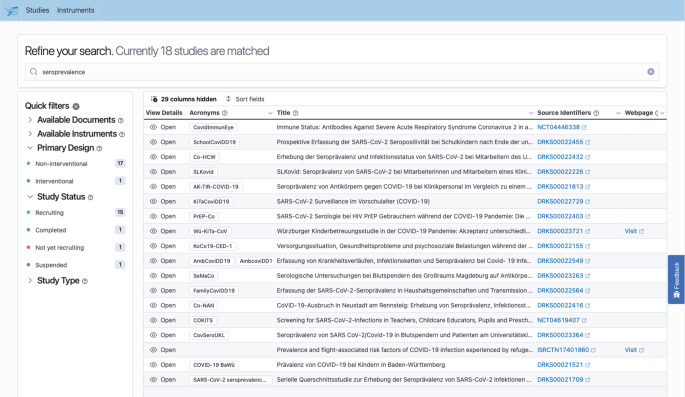

Das zentrale Suchportal (Abb. 2) ist eine als browserbasierte Single Page Application entwickelte Anwendung, welche NutzerInnen einen einfachen Zugang zu Studieninformationen bietet. Zusätzlich werden NutzerInnen über diesen Dienst zu den anderen Webportalen der NFDI4Health Task Force COVID-19 weitergeleitet. Stand Mai 2021 waren 691 Studien eingeschlossen.

Zur Integration der Studieninformationen aus verschiedenen Datenquellen wurde das oben erwähnte Datenmodell [23] verwendet. Dieses erlaubt neben der Beschreibung von Studien auch die Darstellung von anderen Ressourcen mit entsprechenden Metadaten. Dazu zählen Erhebungsinstrumente (z. B. Frage- und Erfassungsbögen) sowie andere Studiendokumente (z. B. Data Dictionaries, Studienprotokolle, Patienteninformationen und Einwilligungserklärungen). Beziehungen zwischen den Ressourcen (Studien, Erhebungsinstrumenten, Dokumenten, Forschenden usw.) können hierarchisch abgebildet werden.

### Dokumentenportal

**Figure 3:**
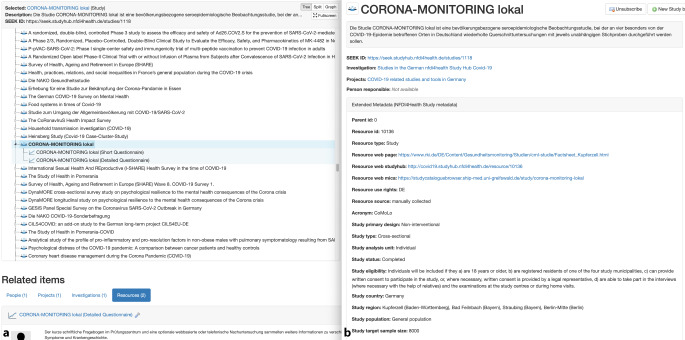

Im Rahmen der europäischen Forschungsdateninitiative FAIRDOM wurde die Plattform „SEEK“ auf Basis der Software Ruby on Rails entwickelt [9, 26]. SEEK wurde im Rahmen der NFDI4Health Task Force COVID-19 an die Anforderungen des Studienportals angepasst. Die Plattform ermöglicht dadurch die Speicherung und Strukturierung vielfältiger Ressourcen und Dokumente von Studien, wie z. B. Erhebungsinstrumente, Standard Operating Procedures (SOPs), Dokumentvorlagen und Studienmanuale, und erlaubt damit einen gebündelten Zugriff auf diese, inklusive Versionsverwaltung, und Möglichkeiten zur Datenpublikation, um Inhalte zitierbar zu machen (Abb. 3). Der Zugang zu den gesammelten Metadaten und Ressourcen der Studien, sofern entsprechende Nutzungsrechte bestehen, ist über eine mit dem zentralen Suchportal verlinkte Benutzeroberfläche (Web-Frontend) sowie über die Nutzung einer programmatischen Schnittstelle (API) möglich, welche zur Suche der Studien auch an das Studienportal angeschlossen ist.

### Instrumentenportal

**Figure 4:**
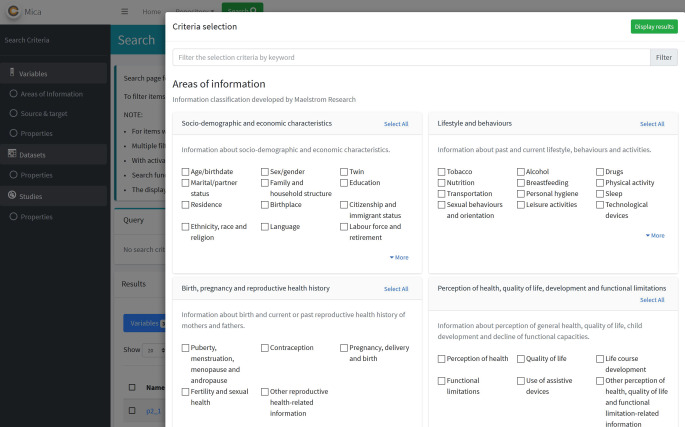

Das Instrumentenportal erleichtert den Zugang zu Inhalten von Erhebungsinstrumenten, z. B. von Survey-Fragebögen und deren Items, d. h. Variablen und zugehörige Wertelisten. Um dies zu ermöglichen, wurden semantische Suchoptionen in den frei verfügbaren Softwareanwendungen OPAL und MICA genutzt [7], die in Java, JavaScript und PHP programmiert sind. Zu diesem Zweck wurden ausgewählte Erhebungsinstrumente für die OPAL-Datenbank aufbereitet und eine semantische Annotation unter Zuhilfenahme der Maelstrom-Taxonomie durchgeführt [19]. Die Taxonomie umfasst 18 Domänen (z. B. soziodemografische und ökonomische Charakteristika, Erkrankungen, nichtpharmakologische Interventionen), die wiederum in 135 Subdomänen (z. B. ICD-Bereiche in der Domäne Erkrankungen) unterteilt sind. Dies ermöglicht eine Suche und Darstellung von Items nach inhaltlichen Kriterien (Abb. 4). Anfang Mai 2021 waren 23 Instrumente mit 3506 Items abgebildet und semantisch annotiert.

Der öffentliche Zugang besteht über den Link [27]. Abgebildet sind sowohl die Fragen und Items als auch deren Antwortkategorien. Eine grafische Übersicht gibt für jedes Instrument Aufschluss über die Anzahl der Items sortiert nach inhaltlichem Bereich, z. B. Lebensstile, Diagnosen und Symptome.

## Semantische Suchmaschine für Preprints

Um einen zentralen Zugriff auf Preprints zu COVID-19 zu schaffen, wurden in einem ersten Schritt Metadaten von den Preprint-Servern medRxiv, bioRxiv, ChemRxiv, ResearchSquare, arXiv und Preprints.org abgefragt und in ein gemeinsames Datenschema konvertiert. Im nächsten Schritt wurden diese Metadaten, z. B. Titel und Abstracts, automatisch mittels Text-Mining [28, 29] unter Zuhilfenahme geeigneter Terminologie indiziert, um die Forschenden durch die semantische Suchfunktionalität bei der Extraktion relevanter COVID-19-Informationen zu unterstützen, beispielsweise basierend auf Konzepten, Autoren, Publikationsdatum oder Quellen. Begleitend wurde eine Terminologie erstellt, um virale SARS-CoV-2-Proteine mithilfe eines wörterbuchbasierten Algorithmus zu erkennen [30]. NutzerInnen stehen die semantischen Suchfunktionalitäten über ein webbasiertes Nutzerinterface und eine Programmierschnittstelle zur Verfügung.

**Figure 5:**
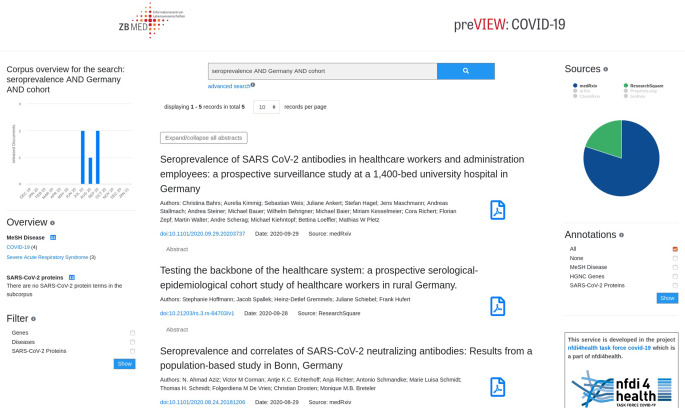

Die semantische Suchmaschine für COVID-19-Preprints, kurz genannt „COVID-19 preVIEW“, umfasst Stand Mai 2021 mehr als 27.000 Preprints aus 6 verschiedenen Servern und ist öffentlich zugänglich unter dem Link [30]. Die Weboberfläche zeigt einen Überblick über die neuesten Publikationen mit Metadaten, wie beispielsweise Titel, DOI und Abstract (Abb. 5). Des Weiteren werden die häufigsten Konzepte für jede Entitätsklasse angezeigt und können ebenfalls zur Suche hinzugefügt werden. Eine Tabelle zeigt die Häufigkeiten der Terme. Weitere Funktionalitäten erleichtern die Informationsextraktion, wie z. B. der Export des Subkorpus im Endnote- oder BibTex-Format.

## Weitere Arbeitsinhalte der NFDI4Health Task Force COVID‑19

Mit dem bisherigen Überblick sind wesentliche, aber nicht alle Arbeitsinhalte der NFDI4Health Task Force COVID-19 dargestellt. So wird als wichtiger Bestandteil der FAIRness von Forschungsdaten die Notwendigkeit einer stärker harmonisierten Herangehensweise an die Datenqualität berücksichtigt. Auf Basis eines bestehenden Datenqualitätskonzeptes [31] und darauf beruhender generischer Analyseroutinen in den Programmiersprachen R [32, 33] und Stata sind Anwendungsbeispiele auch auf COVID-19-Forschungsdaten bezogen, um harmonisierte Datenqualitätsanalysen zu illustrieren. Ein Zugang besteht über die Website der Universitätsmedizin Greifswald [34].

**Figure 6:**
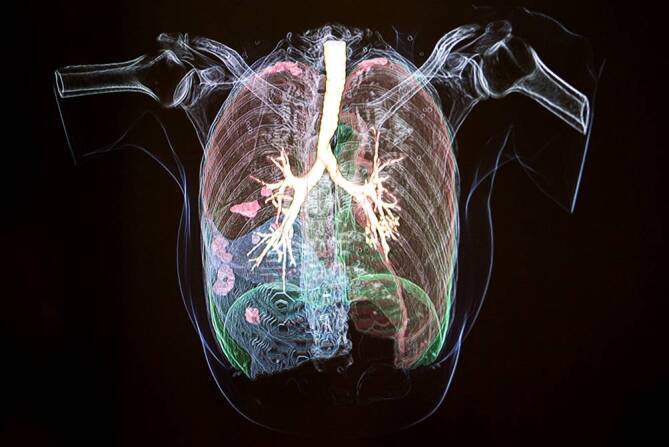

Darüber hinaus wurden in der NFDI4Health Task Force COVID-19 Tools zur Beurteilung der Qualität bei der Bildgebung weiterentwickelt. Dies wurde am Anwendungsbeispiel der Lunge umgesetzt (Abb. 6). Dafür wird die bereits bestehende Plattform Grand Challenge [35] verwendet. Grand Challenge bietet eine Umgebung, um Bilddaten hochzuladen, zu verwalten und Methoden auf die Daten anzuwenden. Bis zu 600 Schnittbilder einer Computertomographie führt die vom Fraunhofer MEVIS, Institut für Digitale Medizin, entwickelte Software zu dreidimensionalen Darstellungen zusammen. Algorithmen für eine automatische Bildanalyse benötigen eine bestimmte Datenqualität, sodass es wichtig ist, auch die Beurteilung der Qualität der zu untersuchenden Daten zu (teil-)automatisieren. Die ausgewählten Daten können zunächst in verschiedenen Ansichten interaktiv betrachtet werden. Relevante Strukturen, wie von COVID-19 betroffene Regionen in der Lunge, werden farbig hervorgehoben. Die Wahrscheinlichkeit einer COVID-19-Erkrankung sowie der Schweregrad des Lungenbefalls werden automatisch berechnet und angezeigt. Die Ergebnisse können als Bericht exportiert und runtergeladen werden. Dabei werden für die Datenschnittstellen standardisierte DICOM-Formate (*Digital Imaging and Communications in Medicine*; [36]) eingesetzt.

Aufbauend auf einschlägigen Publikationen [37–40] wird zudem ein Konzept zur Verknüpfung verschiedener Datenquellen wie primären Forschungsdaten, Krankenhausdaten, Abrechnungsdaten der ambulanten Versorgung, Sequenzierdaten und Bilddaten entwickelt. Dieses Konzept wird durch ein Datenschutzkonzept und eine Vorlage zur Einholung einer informierten Einwilligung ergänzt. Als Herausforderung erweist sich in diesem Teilprojekt der Umgang mit rechtlichen und logistischen Voraussetzungen. Zur Verknüpfung von Routine- und Forschungsdaten sind datenschutzkonforme Einwilligungen und Pseudonymisierungsansätze notwendig. In den meisten Datenquellen werden jedoch nur zweckgebundene Auswertungen durch Einverständnisse und Einwilligungen der ProbandInnen bzw. PatientInnen abgesichert, d. h., eine Verknüpfung verschiedener Datenquellen ist selten vorgesehen. Diese Hürde wurde bereits von der Medizininformatik-Initiative [41] erkannt und ein sogenannter Broad Consent definiert, der eine differenzierte Zustimmung zu einzelnen Verwendungsoptionen abbildet und der durch Datenschutzverantwortliche der Länder akzeptiert wurde. Als zweite, logistische Hürde erwies sich das Fehlen eines übergeordneten bzw. harmonisierten Treuhandkonzepts. Personenidentifizierende und klinische Daten werden durch Dateneigner in lokalen Treuhandstellen verwaltet und unterliegen individuellen Pseudonymisierungsverfahren. Für eine effektivere, übergreifende Nutzung der Daten ist ein übergreifendes Treuhandkonzept erforderlich, das die einzelnen Treuhandstellen miteinander koppelt. Dadurch könnten spezifische Pseudonyme studien- bzw. organisationsübergreifend unter Nutzung spezieller Linkage-Verfahren abgeglichen werden.

## Diskussion

Die NFDI4Health Task Force COVID-19 entwickelt Konzepte, Methoden und Werkzeuge, die eine bessere Übersicht über medizinische, epidemiologische und Public-Health-Forschung zu SARS-CoV‑2 und COVID-19 erlauben. Komplementiert wird dies durch Empfehlungen zum Verknüpfen verschiedener Datenquellen sowie zur harmonisierten Analyse der Datenqualität von Bild- und anderen Forschungsdaten.

Das Studienportal schafft auf Basis neuer Standards zur gemeinsamen Beschreibung von Studien, Erhebungsinstrumenten und weiteren Dokumenten eine Grundlage zur Integration von Informationen aus bisher getrennten Domänen, wie beispielsweise klinischen und epidemiologischen Studien. Durch die studienübergreifend einheitliche semantische Annotation von Items aus COVID-19-Erhebungsinstrumenten besteht im Gegensatz zu anderen Portalen eine leichte Such- und Vergleichbarkeit nach inhaltlichen Kriterien. Zudem wurde mit COVID-19 preVIEW eine semantische Suchmaschine für COVID-19-Preprints entwickelt, die u. a. einen Überblick über die neuesten Publikationen, inklusive Metadaten, ermöglicht.

Die in dieser Arbeit präsentierten Tools und Services befinden sich noch in der Weiterentwicklung und werden durch Feedback der NutzerInnen fortlaufend optimiert. Dies betrifft sowohl die Standards und deren technische Umsetzung als auch die Integration von neuen Inhalten. Zur schnelleren Umsetzung wurde das Datenmodell zur Beschreibung von Studien und Ressourcen zunächst zwar unter Berücksichtigung bestehender Standards, aber doch als eigenständiges Konzept aufgesetzt. Um die Interoperabilität zu anderen Systemen zu gewährleisten und damit auch die Befüllung der Plattform mit Studien und deren Metadaten über entsprechende Schnittstellen weiter automatisieren zu können, ist zukünftig eine Anbindung des Datenmodells an etablierte Standards wichtig, insbesondere an den Interoperabilitätsstandard HL7 FHIR [21]. Auch die Implementation von weltweit gängigen domänenspezifischen Ontologien (Begrifflichkeiten) aus dem medizinischen Bereich, allen voran SNOMED CT [31, 42], wäre wichtig, da sich hierdurch mächtigere semantische Such- und Klassifikationsmöglichkeiten ergäben. Die verwendeten Werkzeuge wie MICA oder SEEK können komplexere Terminologien abbilden; entsprechende Funktionalitäten befinden sich derzeit in Vorbereitung. Weiterhin ist geplant, die vorhandenen Funktionalitäten von SEEK hinsichtlich Strukturierung und Klassifizierung der Studien und Inhalte umfassender zu nutzen.

Technisch wurden im Rahmen der NFDI4Health Task Force COVID-19 mehrere Web-Frontends als Bestandteile eines umfassenden Studienportals verwendet. Dieses Vorgehen bedarf einer weiteren Evaluation, da die Frontends zwar unterschiedliche Schwerpunkte haben, aber dennoch teils überlappende Funktionalitäten besitzen. Deren Zusammenspiel muss im weiteren Projektverlauf unter Berücksichtigung von NutzerInnen-Feedbacks optimiert werden. Eine weitere Herausforderung ist das manuelle Hinzufügen und Kuratieren neuer Metadaten zur Beschreibung von Studien und weiteren Ressourcen. Dies kann nur im Zusammenspiel mit den Verantwortlichen unter der Bedingung knapper zeitlicher Ressourcen umgesetzt werden, was die Akzeptanz reduziert. Daher werden Optionen erprobt, den Prozess der Informationssammlung zu verschlanken, etwa durch Programmierung geeigneter Tools zur Onlineeingabe. Auch die Nutzung etablierter Lizenzmodelle wie Creative Commons zur Klärung der rechtlichen Rahmenbedingungen für eine Nachnutzung von Studiendokumenten in einem zentralen Portal ist nicht trivial. Solche Lizenzmodelle bedürfen noch einer breiteren Verankerung bei den Forschenden.

Die im Rahmen der NFDI4Health Task Force COVID-19 begonnenen Entwicklungen werden im Rahmen der NFDI4Health aufgegriffen und verstetigt. Dies ist sinnvoll, weil die im Zusammenhang mit der aktuellen Pandemie identifizierten Defizite nicht spezifisch für COVID-19-Forschung sind, sondern Forschungsaktivitäten in Public Health, Epidemiologie und klinischen Studien allgemein betreffen. Daher werden Erkenntnisse aus der NFDI4Health Task Force COVID-19 richtungsweisend für weitere Entwicklungen der NFDI4Health sein. Zur Gewährleistung einer hohen Nachhaltigkeit wird auch mit anderen Initiativen auf nationaler und internationaler Ebene eng kooperiert.

## Fazit

Die im Aufbau befindliche Infrastruktur der NFDI4Health Task Force COVID-19 ermöglicht die bessere Auffindbarkeit von Forschung und deren Ergebnissen zu SARS-CoV‑2 und COVID-19 aus Public Health, Epidemiologie und klinischen Studien mit Fokus auf Deutschland. Die im Rahmen der NFDI4Health Task Force COVID-19 umgesetzten Entwicklungen sind über COVID-19 hinaus relevant, da die adressierten Defizite und Herausforderungen generisch für die Auffindbarkeit von und den Umgang mit Forschungsdaten sind.
